# Progress in State Medicine

**Published:** 1902-11-29

**Authors:** 


### Progress in State Medicine.

**Tuberculosis.**—Thornton [1] draws attention to the fact that public benevolence has been more specially devoted to hospitals and sanatoria for consumptives, to the comparative neglect of surgical tuberculosis and scrofula. Fifteen years' experience has convinced him that the knife plays a very subsidiary "rôle" to climatic and general considerations. The wards of hospitals in populous cities are not the most appropriate places in which to treat these cases. Early treatment is essential. This is strongly suggested on examining the cases admitted to the Margate Royal Sea-Bathing Hospital, which show that out of a total of 464 cases discharged during 1901, in which it was possible to get the date of the commencement of the disease, only 85 patients were sent in during the first six months of their illness, and no less than 119 were admitted four or more years after the first signs of tuberculosis became apparent. The question of shelter from wind is of less importance in surgical tuberculosis than it is in cases of lung disease. Early disease of joints and bones benefit most from such treatment, early phthisis in many instances makes remarkable improvement; and many gland cases subside entirely without operation. England is behind other countries in providing seaside hospitals for treating such cases. Weber [2] remarks that it is an important duty to combat the tendency to scrofulosis and to cure the actual disease by all means in our power, this combat being regarded as a branch of the campaign against tuberculosis. The principal causes of scrofulosis are the same as those of tuberculosis. The most powerful remedies are sea air and sea baths, the treatment to extend over several months or years according to the nature of the disease. Hospitals ought to be specially arranged for the cure of scrofulous complaints. They should have large, well-ventilated rooms facing the sea, with broad windows from the ceiling to the floor, which can remain open day and night; they ought to have sheltered terraces or verandahs in front on which patients can lie during the whole day, their beds being wheeled from the room to the terrace through the broad windows. Pavilions of moderate size are preferable to large blocks with three or four stories. There ought to be on the sea-shore sheds open on two sides, which can be turned round according to wind and sun. Children who are permitted to walk ought to play about on the sands, while those who cannot, or ought not to, move must be carried on easy kinds of stretchers to the shore and there remain the whole day if the weather is fair, while in bad weather they are to lie on the sheltered terrace or verandah. There must be good arrangements for bathing in the open sea in summer, and indoor swimming baths of sea-water in winter. Gymnastic apparatus should be provided and used under proper supervision. A specially-arranged boat ought to be attached to the hospital on which, in fair weather patients can be taken to the open sea. These hospitals must be kept open summer and winter, and the duration of the stay of the patients be left solely to the medical attendants. The number of children who require such treatment is very great, but the accommodation in the whole of the United Kingdom is very small. Although it was in England that the necessity for such hospitals was first recognised, other countries have made rapid progress and England has been almost stationary. If the town of Paris considers it a duty to provide seaside treatment to many more than 1,000 scrofulous children of the poor, surely London and other large towns in the United Kingdom ought to do not less. Municipal authorities, county councils, and trade unions, ought to find means for the treatment of poor children; and the well-to-do classes ought to be taxed for the prevention and cure of scrofulosis amongst the children of the poor. Crichton-Browne [3] states that in 1838, out of every 10,000 persons living in England and Wales, 38 died of phthisis, whereas in 1899 of every 10,000 living only 13 died of it. The mortality has therefore diminished by about two thirds in sixty years—the rate of diminution being a slightly increasing one; and with the same rate of diminution for another thirty years, pulmonary consumption will have entirely disappeared. But this decline in mortality will not be maintained without new and special efforts. The beneficent effects of great sanitary reforms and improved conditions of life are, perhaps, approaching exhaustion, and the pressure of modern civilisation is leading to the constant migration of the country population into large towns, with an increase of overcrowding, one of the most prolific causes of consumption. It is to be feared that without some new departure in prevention, the mortality from consumption may again gain on us. Already the diminished death-rate means a saving of 75,000 lives per annum in this country, and instead of waiting for the progressive decline of the next 30 years, fresh efforts are wanted to increase this figure. Referring to overcrowding, he states, that up to 1868, when the report of the Royal Commission on the sanitary state of the army was published, our barracks were shockingly overcrowded, and the mortality from phthisis amongst the troops was appalling. But immediately the allowance of cubic space per man in the barracks was increased, and the ventilation attended to, there was an instantaneous and marked fall in the phthisis mortality. It dropped very rapidly from 11 to 3 per annum per 1,000 strength. Certain diseases, such as measles, typhoid fever, and diabetes, often leave behind a strong proclivity to tuberculous disease, and certain persistent postures of the body, involving stooping, contraction of the chest, and imperfect expansion of the lungs, whether imposed by education, industrial pursuits, or sheer indolence, also predispose to it. Of all patients between the ages of 25 and 75 dying in hospitals in London, 20 per cent.

show signs of old tubercular lesions in the lungs which have healed up. Even in fatal cases attempts at healing are found going on in the lung side by side with extensions of the disease. The increased knowledge of the tubercle bacillus provides fresh weapons wherewith to fight it. Sanatorium treatment has for its object the increase of the vital resistance of the patient, and the heightening of his or her immunity to the slowly progressing disease. This treatment should be carried out under constant medical supervision. It is not merely treatment but a discipline, and the patient to derive full benefit from it must be dominated by his doctor for the time being. It seems clear that in cases of consumption in the first stage submitted to thorough sanatorium treatment, 50 per cent will be permanently cured, and a marked improvement made in another 25 per cent. of cases. Broadbent[4] emphasizes that it is not sufficient to try to prevent the dissemination of consumption by destroying sputum, but it must be followed up wherever its existence was suspected by disinfection and ventilation. England is not yet ready for compulsory notification. At present voluntary notification must be relied on. One form of compulsory notification ought to exist—that is after death. The notification should be compulsory, not only to the registrar, but to the Health Authorities, of all deaths from consumption, so that the rooms inhabited by the deceased shall not become a source of infection to those who succeed.

[1] Turberculosis, April, 1902. [2] Ibid. [3] Ibid. [4] Sanit. Rec., May 1, 1902.

